# Physico-chemical pretreatment and fungal biotreatment for park wastes and cattle dung for biogas production

**DOI:** 10.1186/s40064-015-1466-9

**Published:** 2015-11-20

**Authors:** Sameh S. Ali, Jianzhong Sun

**Affiliations:** Botany Department, Faculty of Science, Tanta University, 31527 Tanta, Egypt; Biofuels Institute, Jiangsu University, Zhenjiang, 212013 China

**Keywords:** Cellulosic biomass, Pretreatment, Biogas, Methane

## Abstract

With the rising demand for renewable energy and environmental protection, anaerobic digestion of biogas technology has attracted considerable attention within the scientific community. The effect of physico-chemical pretreatment on cellulose degradation followed by fungal treatment by *Aspergillus terreus* and *Trichoderma viride* to treat cellulosic biomass for enhancing its digestibility was investigated. The tested substrate was digested with and without physical, chemical, and biological treatment. Fresh leaves, dry leaves and cattle dung were characterized by a total solids content 35, 84 and 17 %, volatile solids content 81.2, 59.49 and 64.5 % and C/N ratio 31, 45.4 and 13.6, respectively. Biogas total volume was determined using water replacement technique, while methane volume was determined using precipitation of CO_2_ in 20 % NaOH solution. Pretreatment steps were carried out by using mechanical and chemical pretreatments using 2.5 % NaOH mixed with 2.5 % NH_4_OH for 15 days, followed by biological treatment of *A. terreus* and *T. viride*. The potential of pretreatment of substrate was studied at regular intervals of 0, 7, 14, 21, 28, 35, 42, 49, 56, 63 and 70 days determining the change in chemical and physical compositions of used substrates. Biogas production was 102.6 and 125.9 L/KgVS from untreated and pretreated substrate, respectively. On the other hand, methane production was 61.4 and 79.8 L/KgVS from untreated and pretreated substrate, respectively. In conclusion, Physical (milling), chemical (NaOH and NH_4_OH) pretreatment in addition to fungal (*A. terreus* and *T. viride*) treatment for the tested substrate prior to AD was an efficient process for improvement of biogas and methane production.

**Electronic supplementary material:**

The online version of this article (doi:10.1186/s40064-015-1466-9) contains supplementary material, which is available to authorized users.

## Background

The consumption of renewable energy is dramatically increasing, along with energy security concerns, efforts to mitigate the environmental impact of conventional fuels, and improvements in living standards and renewable technologies. Bioenergy can play a central role in promoting renewable alternatives. In fact, bioenergy is estimated to be the fourth largest energy resource in the world (Chen and Lee 2014) due to its renewable and widely applicable characteristics and its abundance. Forestry resources, agricultural resources, sewage and industrial organic wastewater, municipal solid wastes, live-stock and poultry dung and biogas are major categories for use. Biogas, which is generally referring to gas from anaerobic digestion (AD) units, is a promising means of addressing global energy needs and providing multiple environmental benefits (Jiang et al. 2011). Biogas not only significantly reduces the costs of treating waste (Holm-Nielsen et al. 2009) but also has a relatively low feedstock cost. In addition, biogas has a lower sale price compared with diesel and petrol.

Biogas is generated from a digestion process under anaerobic conditions whose application is rapidly emerging as a viable means for providing continuous power generation. The AD cycle represents an integrated system of a physiological process of microbial and energy metabolism, as well as raw materials processing under specific conditions to produce biogas which is composed of methane and carbon dioxide and trace gases (Wu 2012). The pretreatment of wastes before AD is considered an effective method to improve biodegradability and biogas production from lignocellulosic materials by speeding up the hydrolysis step (Chen et al. 2005). Usual pretreatment methods including physical, chemical and biological may be used singly or in combinations (Sari and Budiyono 2014). Lignocellulose wastes mainly include crop residues and logging residues with crop residues making up the majority. For China, more than 800 million metric tons of waste agricultural straw is produced per year (Bi et al. 2009). However, this waste cannot be digested by itself due to recalcitrant materials (lignin, cellulose and hemi-cellulose) that result in low biodegradation and poor digestion performance; thus, extra accumulative measures are needed to start the digestive process such as pre-treatment and inoculums (Zhang et al. 2011).

Physical pretreatments were carried out to break down the cellular structure of biomass and to increase the specific surface area. This provided the chance for the lytic action of bacterial enzymes and reduction of viscosity in digesters, which is particularly important for cellulosic substrates (Schell and Harwood 1994). Chemical reagents are predominantly used for pretreatment of lignocelluloses materials due to their low-cost and high efficacy. By changing the properties of raw material, e.g., increasing the surface area, removing or dissolving lignin and hemicellulose, and reducing the crystallinity of cellulose, chemical reagents make lignocellulosic biomass more biodegradable and accessible to anaerobic microorganisms (Sun et al. 2014; Mao et al. 2015).

Microbiological pretreatment can speed up the degradation rate of substrates in anaerobic digestion. Bacterial and fungal cellulose-, hemicellulose- and starch-degrading enzymes are responsible for enhancing the substrate digestibility (Muñoz et al. 2014). However, the microbial community is sensitive to variations in the operating conditions applied. Thus, the AD process, if improperly managed, would become unstable and result in reduced biogas production. Although previous studies have discussed AD development, most focused on only one aspect (such as technology, mechanism, factors affecting efficiency, etc.) to minimize this instability (Munk et al. 2010) or on one substrate (such as livestock manure, urban solid waste, food waste, crop straw, etc.). Fungi, particularly those that attack lignin, are mainly used in the pretreatment of lignocellulosic biomass for biogas production. Several fungi classes, including brown-rot, white-rot and soft-rot fungi (i.e., *Ceriporiopsis subvermispora*, *Auricularia auricula*-*judae*, *Trichoderma reesei*), and basidiomycetes (e.g., *Ischnoderma resinosum* and *Fomitella fraxinea*) have been used for pretreatment with white-rot fungi being the most effective through the action of lignin-degrading enzymes (e.g., peroxidases and laccase) (Zheng et al. 2014). After fungal pretreatment, a 5–15 % increase in the methane yield was obtained (Mackuľak et al. 2012; Sun et al. 2014). The aim of this study is to improve the performance of anaerobic digestion of lignocellulosic park wastes and cattle dung, by applying physico-chemical pretreatments and fungal fungal treatment. In addition, daily and cumulative production of biogas, CH_4_ and CO_2_ is evaluated for both untreated and pretreated. Beside, study of the changes in different parameters that control the anaerobic digestion process.

## Methods

### Procurement of the materials

Samples of Park wastes (fresh and dry leaves) were collected from Faculty of Science at Tanta University parks. The fresh and dried leaves were chopped to 3–4 cm and were stored in polythene bags at room temperature. Cattle dung sample of the various microbial inocula for AD had been collected from a farm at El-Hamrawy village in Kafr El-Sheikh Governorate, Egypt and placed in sealed plastic bags and then frozen at −20 °C, to retain microbial viability in the collected sample (Williams and Withers 2012).

### Physical pretreatment

Before mixing with cattle dung, mechanical pretreatment was performed on the collected park wastes (fresh and dried leaves), by using a cutting mill and then sieved with 2 mm screen (Bochmann and Montgomery 2013) In order to achieve a squeezing for the cellular structure of park wastes for the pretreatment prior to anaerobic digestion.

### Chemical pretreatment

Lignocellulosic materials are resistant to hydrolysis due to their structure and composition. Alkali addition causes swelling of lignocelluloses and partial lignin solubilisation (Kong et al., 1992). Park wastes were soaked in combination of 2.5 % NaOH and 2.5 % NH_4_OH for 15 days in a closed plastic container to improve delignification levels (Elumalai et al. 2014). After chemical pretreatment step, pH was adjusted using dilute 1 N HCl to achieve a suitable medium for the biological pretreatment and fungal growth (Alvarez et al. 2013).

### Biological treatment

#### Isolation and identification of fungi

Fungi were isolated from an old digester slurry (cattle dung and food waste mixture) using a tenfold serial dilution-plating technique on potato dextrose agar (PDA) plates into which 30 µg of chloramphenicol was added. The plates were incubated at 25 °C. The cultures were observed daily and fungal growth was subcultured onto fresh PDA plates until pure isolates were obtained. The plates were identified macroscopically and microscopically using colony color, type, texture, shape and growth pattern according to the detailed drawing of the features and identification manual and guides of Gilman (1959), Booth (1971), Moubasher (1993) and Blackwell (2011). The isolated fungi were maintained on potato dextrose agar (PDA) slant and stored at 4 °C.

#### Preparation of inocula

Inoculum suspensions were prepared from fresh, mature cultures grown on PDA and Sabouraud agar. The colonies were covered with 5 mL of distilled water. Tween 20 (5 %) was added to facilitate inoculum preparation. The mixture was collected in a sterile tube with vortexing for 15 s (Petrikkou et al. 2001).

#### Preparation of starter culture

In order to obtain biogas and methane production, the method of Maramba (1978) was employed. Cattle dung, which is well known to contain methanogenic consortia, will be used to start the inoculation of the digester. The starter material was prepared 20 days before the start of charging fresh substrate in the digester. A volume of 500 mL of starter was prepared at 1:1 ratio (water to cattle dung) and placed in a closed plastic bottle connected with a submerged tube, so all the gas can come out without air entrance to the drum.

### Experiment set-up

**Fig. 1:**
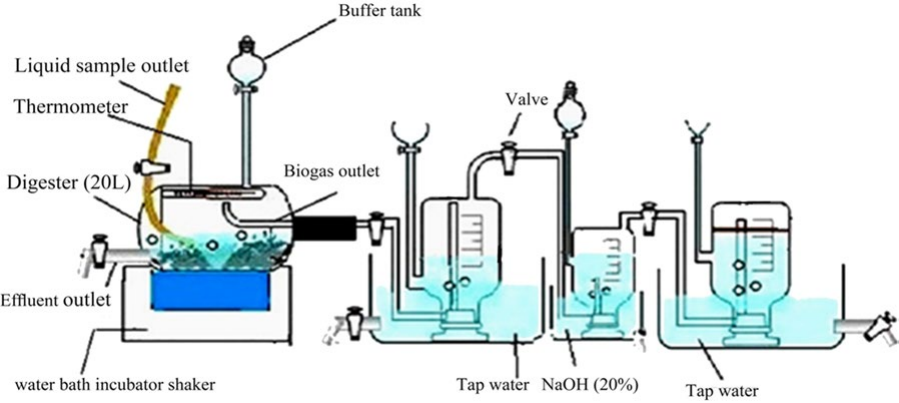
Schematic biogas batch digester

Two digesters each contained a mixture of 125 g of fresh leaves, 125 g of dried leaves and 250 g of cattle dung. The substrate in the first digester was milled to 2 mm then pretreated with alkaline solution (2.5 % NaOH mixed with 2.5 % NH_4_OH) and the pH was adjusted by using diluted 1 N HCl to be a suitable medium (pH 7.1) for biological treatment by two isolated and identified fungi (*Aspergillus terreus* and *Trichoderma viride*) isolated from the old digester slurry. The second digester was left without any pretreatment as a control. Fungal treatment of park wastes was kept at 25 °C for 7 days followed by incubation of the both digesters for 70 days on incubator shaker (35 °C, 120 rpm) to assist in the mixing process as shown in Fig. 1. Physico-chemical analyses samples were collected from effluent outlet at the bottom of the digester at regular intervals of 0, 7, 14, 21, 28, 35, 42, 49, 56, 63 and 70 to determine pH, EC and C/N ratio.

### Biogas, methane and carbon dioxide volume

Daily methane volume was determined using a system developed in our laboratory of Microbiology at Faculty of Science, Tanta University. The system consists of four units connected together using connecting pipelines, as shown in Fig. 1. The first unit contains a simple designed batch plastic digester equipped with biogas outlet, solid sample outlet, thermometer and valve spout effluent outlet. The second unit is an application for water replacement technique which is used to measure total volume of biogas. Ergüder et al. (2000) reported that the gas production in batch reactors was determined by a water displacement device. The third unit used to determine the volume of methane and carbon dioxide gases by passing produced biogas in 20 % NaOH. After biogas elevation, the container shaken and left for 30 min to measure the methane volume according to (Manilla et al. 2001) and Wojciech (2012) who reported that sodium hydroxide (NaOH) reacts with carbon dioxide (CO_2_) through chemical processes. When the produced biogas passes through sodium hydroxide, sodium bicarbonate (NaHCO_3_) is formed according to Eq. (1). In this way, carbon dioxide and water moisture were removed from the entire gas.

1 $${\text{NaOH}} \left( {aq} \right) + {\text{CO}}_{ 2} \left( g \right) \to {\text{NaHCO}}_{ 3} \left( {\text{aq}} \right)$$

The content of CH_4_ in biogas was determined as follows: A known volume of the headspace gas was produced in a two liter container to determine the total volume of biogas by water replacement technique (V_B_), and it was injected into another container which contained 20 % NaOH solution. This container was shaken manually for 3–4 min so that all the CO_2_ could be absorbed in the concentrated NaOH solution, then it was left for 30 min. The volume of remaining gas (V_M_) refers to the produced methane volume. Hence, the volume of methane and carbon dioxide (V_C_) can be calculated according to Eq. (2) (Ergüder et al. 2000; Tippayawong and Thanompongchart 2010; Drosg et al. 2013).

2 $$V_{B = } V_{M} + V_{C}$$

The fourth unit used to collect and store all purified methane gas.

### Physico-chemical analysis

The physico-chemical properties of park wastes before and through anaerobic digestion process were implemented at Ministry of Agriculture and land reclamation laboratories in Gharbia in addition to Kafr El-Sheikh University central laboratory of environmental studies (KUCLES).

#### Total solids (TS) and volatile solids (VS)

Analyses of samples were carried out using APHA standard methods (APHA 1995) by taking a known weigh of sample in dried aluminum container. At the beginning, the weight of empty containers alone and containers with samples together was determined. The containers were placed in a furnace at 105 °C for 12 h to evaporate water for total solids (TS) calculation. Weight of the containers was determined. For volatile solids (VS) calculations, the contents were put in another furnace at 550 °C for 3 h. The weight was then recorded. Mathematical formulas for TS and the VS calculation are shown below. Organic matter (OM) is the percentage of VS/TS.

3 $${\text{TS}} = \frac{{{\text{A}} - {\text{B}}}}{{{\text{C}} - {\text{B}}}}$$

4 $${\text{VS}} = \frac{{\left( {{\text{B}} + {\text{A}}} \right) - ({\text{B}} + {\text{D}})}}{{({\text{B}} - {\text{A}})}} - {\text{B}}$$

A: weight of dried sample after 12 h at 105 °C, B: weight of container, C: weight of wet sample, D: weight of burnt sample after 3 h at 550 °C.

#### pH measurement

A mass of 10 g of digester slurry was added to a sample cup. The sample was mixed with water (1:1 v/v). The slurry allowed setting, after vigorous stirring, for 1 h at room temperature. Then pH was measured by pH meter (Miller and Kissel 2010).

#### Electric conductivity (EC)

A weight of 5 g air dried sample was dissolved in 50 mL distilled water (1:10 w/v) and shaken on an orbital shaker for 40 min. Then supernatant was taken and EC of the supernatant was recorded using a pen type Total dissolved solids (TDS) meter (Peters et al. 2003). The results were expressed in dSm^−1^ according to Eq. 5.

5 $${\text{TDS in mgL}}^{ - 1} \left( {\text{ppm}} \right) \, = { 640 } \times {\text{ EC }}\left( {{\text{dSm}}^{ - 1} } \right)$$

#### Total organic carbon (TOC)

Total organic carbon in the tested substrates was determined according to Page et al. (1982) by multiplying Organic matter percentage in 0.58.

#### Total Kjeldah nitrogen (TKN)

For determination of organic and the inorganic forms of nitrogen, Total Kjeldahl Nitrogen (TKN) was analyzed by kjeldahl digestion method (APHA 1992). The substrate sample (1 g) was digested at 400 °C for 60–90 min. by adding 12 mL of concentrated sulphuric acid. For raising the boiling point, 3.5 g potassium sulfate (K_2_SO_4_) was added. In addition to 0.4 g of copper sulfate (CuSO_4_) was added as a catalyst. The tubes are left to cool off until the disappearance of white fumes. Subsequently, 50 ml sodium hydroxide was automatically added to the sample for convert NH_4_^+^ to NH_3_. The sample was distilled and condensed into 10 mL of boric acid solution, and the content was determined by acid–base titration. The titration endpoint was situated around pH 5.3 with color change visualization from green to blue/purple and by addition of methyl red methylene blue indicator.

### Statistical analysis

Results are presented as the mean ± standard deviation (SD) from three replicates. The statistical analyses were carried out using SAS (v 6. 12). Data obtained were analyzed statistically to determine the degree of significance using one way analysis of variance (ANOVA) and *t* test at probability level p ≤ 0.05.

## Results

### Substrate characterization

As shown in Additional file 1: Table S1, the park wastes (fresh and dry leaves) and cattle dung characterization revealed that the dried leaves had the highest TS content (84 %) and the highest value of C/N (45.4). On the other hand, cattle dung had low TS content and C/N ratio representing 17 % and 13.6, respectively. Fresh leaves contained the highest VS and TOC representing 81.2 % and 47.1 %, respectively. The highest percentage of TNK was recorded for cattle dung (2.8 %).

### Biogas and methane production

The optimization conditions for biogas and methane production from the used substrate were investigated through two pretreatment processes including physical (milling) and chemical (2.5 % NaOH mixed with 2.5 % NH_4_OH for 15 days) followed by fungal treatment using *Aspergillus terreus* and *Trichoderma viride* for 7 days. Biogas production and process parameters were recorded during 70 days of retention time.

**Fig. 2:**
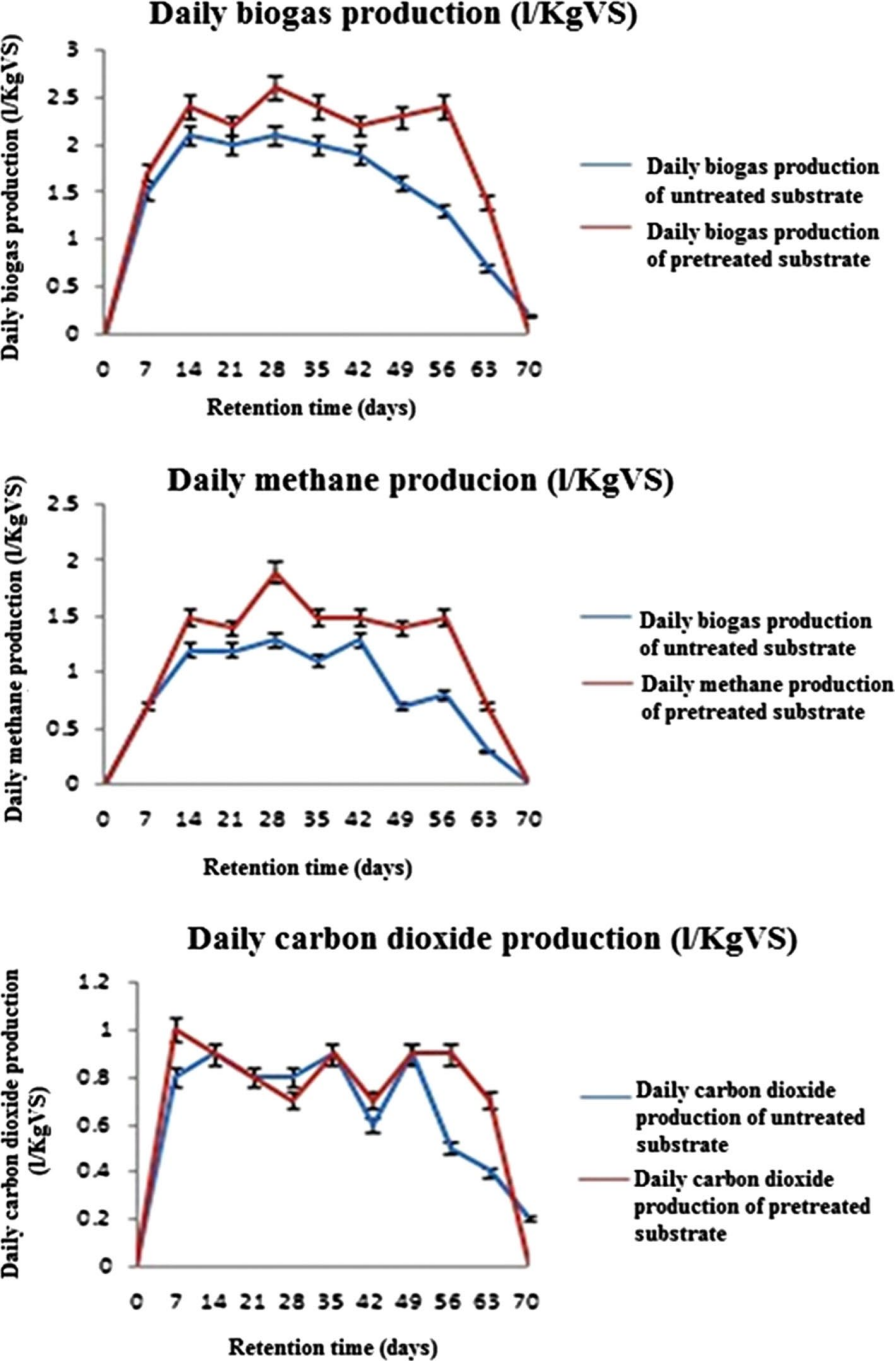
Daily biogas, methane and carbon dioxide production

**Fig. 3:**
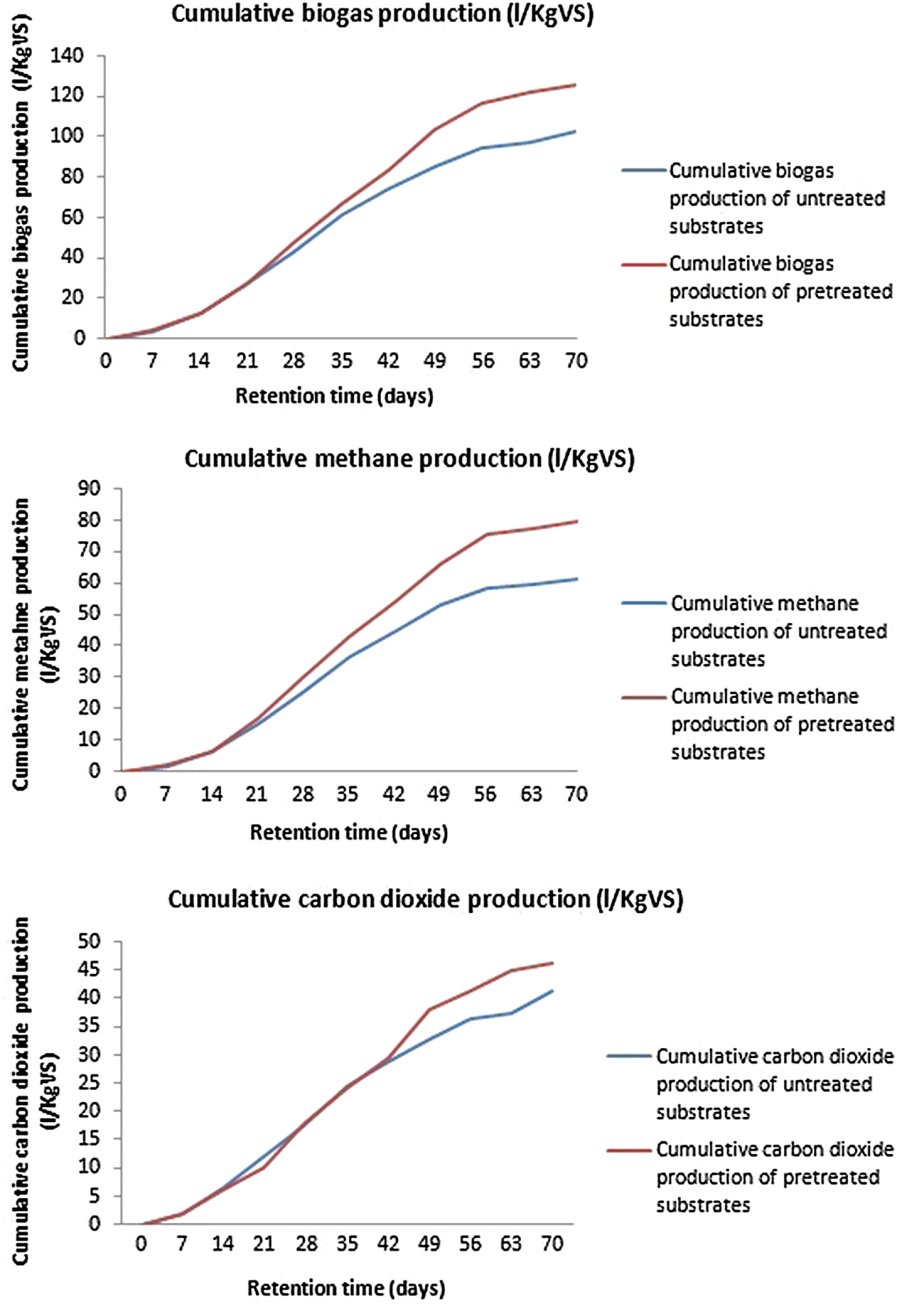
Cumulative biogas, methane and carbon dioxide production

Biogas and CH_4_ yields of the pretreated and untreated substrate were measured. The daily and accumulative productions of biogas and CH_4_ obtained during 70 days of digestion from untreated and pretreated substrate were shown in Additional file 1: Table S2 and in Figs. 2 and 3. Daily biogas and CH_4_ production yields from the substrate were improved by the three pretreatment stages. The highest biogas and CH_4_ yield of 2.6 and 1.9 L/KgVS, respectively was obtained from pretreated substrate in the 28^th^ day compared to untreated substrate. On the other hand, the cumulative production of biogas for untreated substrate was 102.6 L/KgVS which elevated to 125.9 L/KgVS in case of pretreated substrate which showed 22.7 % improvement compared to the biogas yield from untreated substrate. Cumulative CH_4_ production of pretreated and untreated substrate was 79.8 and 61.4 L/KgVS, respectively with 30 % improvement compared to the methane yield from untreated substrate. CO_2_ showed a reduction rate after pretreatment quantified by 11.9 %.

Statistical analysis of biogas, methane and CO_2_ production of untreated and pretreated substrate was shown in Additional file 1: Table S2. Substrate pretreatment had a significant improvement effect on the biogas and methane production after the 14th day; while being insignificant to that till the 56th day, and this showed the stability in the biogas and methane production through AD period. Beside, CO_2_ production showed significant decreasing values after 14th day and insignificant decrease from the 28th till 56th day.

### pH, EC and C/N

**Fig. 4:**
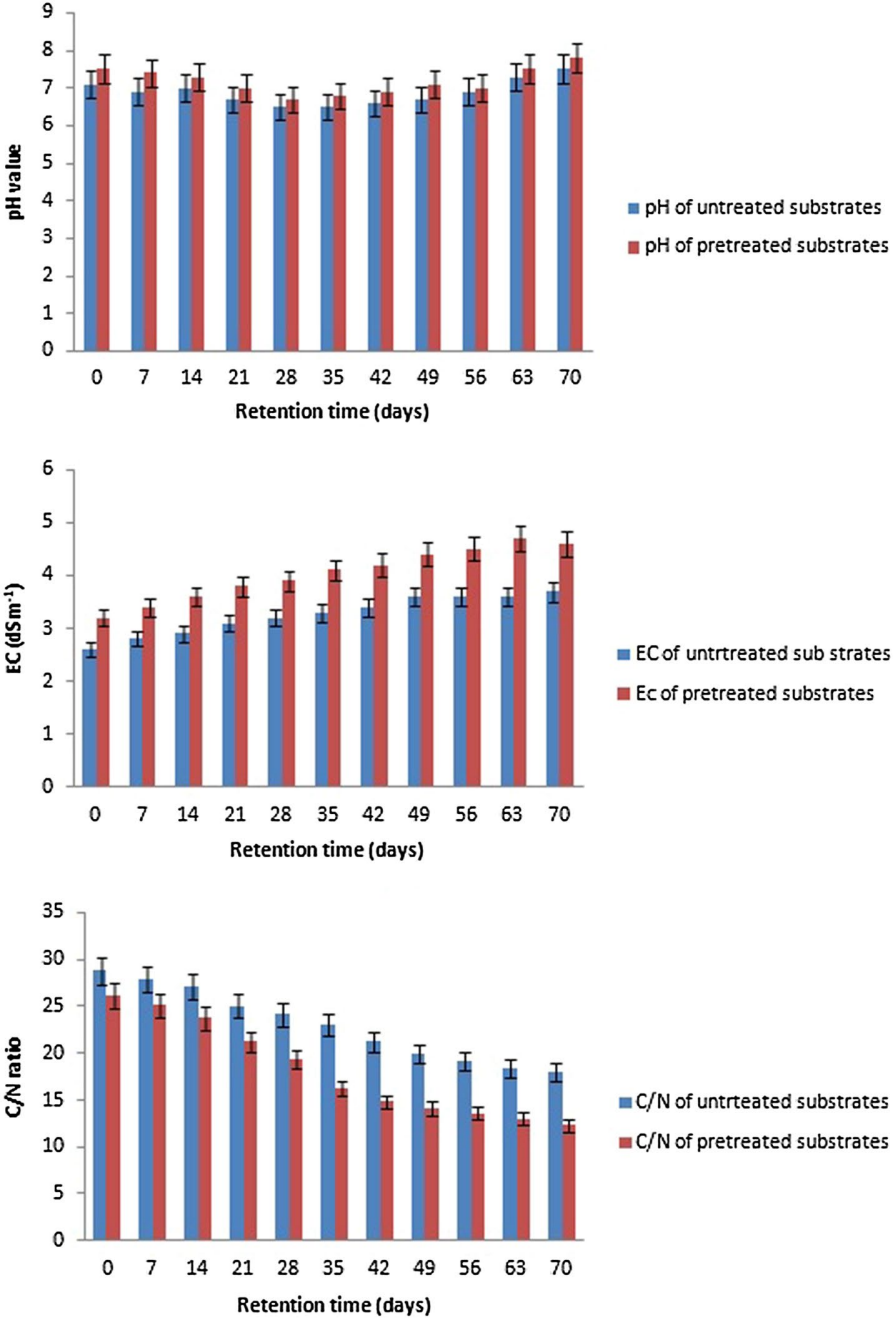
Changes in pH, EC and C/N ratio through AD process

The results of pH, EC and C/N of the untreated and pretreated substrate are shown in Additional file 1: Table S3 and Fig. 4.

Insignificant decrease in pH values of untreated substrate till the 2nd week of AD followed by significant decreasing till the 8th week. An increase in pH values was observed again at the beginning of the 8th week till the 10th week for both untreated and pretreated substrate. The changes in Electric conductivity (EC) of untreated substrate were observed (2.6–3.7 dSm^−1^) and showed directly proportional increasing along with the retention time. Moreover, EC values from the substrate were improved by its pretreatment (3.2–4.6 dSm^−1^). Statistical analysis showed significant changes in EC for both untreated and pretreated substrate. However, insignificant increase from the 56th day till the 70th day was recorded for the pretreated substrate. Additional file 1: Table S3 and Fig. 4, represented a significant change in C/N ratio during AD process for 70 days. Pretreatment of the substrate improved C/N in the first day to be 26.1 compared to untreated substrate (28.8), and decreased to be 12.3 at the end of AD time for pretreated substrate. In the same concern, a significant decreasing in C/N for untreated substrate was observed through the period of retention time to be 18 at the end of AD time.

## Discussion

Lignocelluloses are basically composed of carbohydrates (cellulose and hemicelluloses), lignin, and extraneous materials (Karimi et al. 2013). However, the compact crystalline structure where lignin physically protects the cellulose and hemicelluloses parts makes these materials more resistant to anaerobic digestion (Mosier et al. 2005). In the anaerobic digestion process, if a substrate is well enclosed in lignin structures, the type of disintegration of the substrate becomes important. The structure should be disrupted or defibrated rather than cut, because they are refractory to decomposition under anaerobic conditions (Pesta 2007). Without prior treatments, a slow hydrolysis might occur and biogas production could become low with a long retention time required to produce sufficient amount of biogas (Yu and Schanbacher 2010). In the present study, the used substrate was soaked in alkaline solution (2.5 % NaOH mixed with 2.5 % NH_4_OH) followed by fungal treatment for 7 days. Zuo et al. (2012) showed that a novel soaking pretreatment (NaOH and aqueous-ammonia) for corn stover was developed to remove lignin, swell the biomass, and improve enzymatic digestibility. Furthermore, alkalis help prevent decreases in pH during the acidogenesis process and increasing the efficiency of methanogenesis (Mao et al. 2015). Taherzadeh and Karimi (2008) showed that in alkaline pretreatment the biomass is soaked by alkaline solution at relatively low temperature for a certain amount of time. Zhang et al. (2011) compared the results of biogas production from corn straw which was subjected to 8 % NaOH, 5 % ammonia and 4 % urea pre-treatments at an ambient temperature for 20 days prior to anaerobic digestion. He et al. (2008) showed a significant increase in biogas yield in batch tests using rice straw pretreated with 6 % solid NaOH for 3 weeks at ambient temperature. Ghosh and Bhattacharyya (1999) studied white-rot fungi and reported their ability on complete lignin degradation, and their application has been suggested for partial delignification to increase digestibility. In the same concern, Song et al. (2013) reported that the pretreatment with white-rot fungi can effectively remove lignin and decompose the biomass structure to enhance subsequent enzymatic hydrolysis. As well as, alkali pretreatment led to delignification of lignocellulosic fibers and the substrate became easier to be biodegraded. Sambusiti et al. (2012) reported that recombination of different pretreatment methods led to a solubilization of holocellulose and lignin. Ishola et al. (2014) indicated the ability of recombination between physical, chemical and biological pretreatments to degrade cellulosic biomass and increase its digestibility and its effect on biogas and methane production.

In the current study, the methane yield was enhanced by 30 % after the use of fungal (*A. terreus* and *T. viride*) and alkaline pretreatment (2.5 % NaOH mixed with 2.5 % NH_4_OH). Thus, the pretreatment used in this work was effective compared to Liew et al. (2011) who showed that the methane yield increased by 20 % using 3.5 % NaOH on fallen leaves. Zheng et al. (2009) and Yu et al. (2014) reported that the simultaneous alkaline treatment not only helped to improve the digestibility of park wastes but also increased the buffering capacity of the digester to maintain suitable pH, thus leading to significant increase in biogas and methane yield compared with no or low alkaline loadings. The biological pretreatment has been extensively studied due to its several advantages, such as non-toxicity, environmental friendly, no chemical requirement, mild environmental conditions and low energy requirements. However, long pretreatment time is needed because the rate of the biological hydrolysis is usually very low, which is the main disadvantage of biological pretreatment methods (Taherzadeh and Karimi 2008). The obtained results revealed the improved biogas production (22.7 %) by chemically and biologically substrate pretreatment (125.9 L/KgVS) compared to untreated substrate (102.6 L/KgVS). In addition, CH_4_ production of pretreated substrate (79.8 L/KgVS) was improved than untreated one (61.4 L/KgVS) by 30 %. On the other hand, carbon dioxide production was reduced by 11.9 % after pretreatment. The present study showed the changes in pH values of untreated substrate from 7.1 to 7.5 after 70 days, and from 7.5 to 7.8 for pretreated substrate. The pH value of the AD substratum influences the growth of methanogenic microorganisms and the dissociation of some compounds relevant for the AD process, i.e. ammonia, hydrogen sulphide and organic acids (Al Seadi et al. 2008). Methane formation takes place within a relatively narrow pH range, between 5.5 and 8.5 ca., with an optimal range of between 7.0 and 8.0 for most methanogens (Al Seadi et al. 2008). Ho et al. (2013) stated that the drop occurred in pH values was due to the formation of volatile fatty acids after anaerobic digestion process, which caused a drop in pH value to turn the conditions favourable to methanogenic bacteria which consumed acetic acid and raise pH value again. Moreover, EC values of untreated substrate varied from 2.6 to 3.7 dSm^−1^. However, in pretreated substrate EC reached to 4.6 dSm^−1^ by the end of anaerobic digestion. The gradual increase in EC values might be due to high concentration of nutrient ions released during the organic matter mineralization. Bakili et al. (2014) and Okoroigwe et al. (2014) reported that the C/N ratio must range from 20 to 30 to make a nutritional balance for microorganisms inside the digester. The current results showed that the C/N ratio changed from 28.8 (untreated substrate) to 26.1 after physical, chemical pretreatment in addition to fungal (*A. terreus* and *T. viride*) treatment, revealing the effect of these pretreatment steps in improvement of C/N ratio. The C/N ratio of untreated and pretreated substrate showed a gradual decrease C/N values along with retention time due to bacterial activity that consumes total organic carbon as well as using a small amount of nitrogen (Tchobanoglous et al. 1993). The content of organic matter and organic carbon decreased because of the physico-chemical pretreatment and fungal activities which can degrade cellulose and lignin and hence increase biogas production. Weiland (2010) reported that a ratio of around 30 is favourable for microbial cell metabolism. But lower C/N ratio than 10–15 leads to ammonium releasing and inhibition of bacterial growth. This explains, in the present study, the decreasing of biogas production after lowering C/N ratio through anaerobic digestion. This decreasing in C/N ratio may affect methanogenic bacteria and thus decreasing in methane yield.

## Conclusion

Physical (milling), chemical (NaOH and NH4OH) pretreatment in addition to fungal (*A. terreus* and *T. viride*) treatment for park wastes and cattle dung substrate prior to AD was an efficient process for improvement of biogas and methane production. Moreover, further work is needed to ensure that the recycling of treated and untreated lignocellulosic wastes results in the improvement of agriculture.

## Additional file

10.1186/s40064-015-1466-9 In the supplemental material section results from park wastes and cattle dung characterization as well as statistical analysis of biogas, methane and CO_2_ daily and cumulative production of untreated and pretreated substrate in addition to statistical analysis of pH, EC and C/N of the untreated and pretreated substrate are presented.
