# Supplementary material for: Physico-chemical pretreatment and fungal biotreatment for park wastes and cattle dung for biogas production
Source: Springerplus. 2015 Nov 20;4:712. doi: 10.1186/s40064-015-1466-9 (PMC4654729; doi:10.1186/s40064-015-1466-9)
Supplement: Supplementary file 1 — 10.1186/s40064-015-1466-9 In the supplemental material section results from park wastes and cattle dung characterization as well as statistical analysis of biogas, methane and CO2 daily and cumulative production of untreated and pretreated substrate in addition to statistical analysis of pH, EC and C/N of the untreated and pretreated substrate are presented. [file 40064_2015_1466_MOESM1_ESM.docx]

**TABLES**

**Table S1.** Substrate characterization

| Constituents | Cattle dung  (Starter) | Park wastes | |
| --- | --- | --- | --- |
|  |  | **Fresh leaves** | **Dry leaves** |
| Total solid (%) | 17.0 | 35.0 | 84.0 |
| Volatile solid (%) | 64.5 | 81.2 | 59.5 |
| Organic carbon (%) | 37.4 | 47.1 | 34.5 |
| Total nitrogen ( %) | 2.8 | 1.5 | 0.8 |
| C/N | 13.6 | 31.0 | 45.4 |
| pH | 7.2 | 6.8 | 5.8 |
| EC | 1.33 | 2.9 | 2.1 |

**Table S2.** Daily and cumulative production of biogas, methane and CO2 in untreated and pretreated substrate

| Cumulative production (l/KgVS) | | | | | | Daily production  (l/KgVS) | | | | | | RT  (days) |
| --- | --- | --- | --- | --- | --- | --- | --- | --- | --- | --- | --- | --- |
| Pretreated | | | Untreated | | | Pretreated | | | Untreated | | |  |
| CO_2_ | CH_4_ | Biogas | CO_2_ | CH_4_ | Biogas | CO_2_ | CH_4_ | Biogas | CO_2_ | CH_4_ | Biogas |  |
| 0.0±0.00^a^ | 0.0±0.00^a^ | 0.00±0.00^a^ | 0.0±0.00^a^ | 0.0±0.00^a^ | 0.00±0.00^a^ | 0.0±0.00^a^ | 0.0±0.00^a^ | 0.00±0.00^a^ | 0.0±0.00^a^ | 0.0±0.00^a^ | 0.00±0.00^a^ | 0 |
| ±0.26^b^2.1 | ±0.95^b^2.1 | ±1.11^b^4.2 | ±0.05^b^2.1 | ±0.08^b^1.8 | ±1.05^b^3.9 | ±0.05^b^1.0 | ±0.08^b^0.7 | ±0.11^b^1.7 | ±0.08^b^0.8 | ±0.03^b^0.7 | ±0.10^b^1.5 | 7 |
| 6.2±0.75^c^ | 6.4±1.13^c^ | 12.6±0.39^c^ | 6.6±0.35^c^ | 6.3±0.22^c^ | 12.9±0.35^c^ | 0.9±0.10^c^ | 1.5±0.17^c^ | 2.4±0.26^c^ | 0.9±0.13^c^ | 1.2±0.05^c^ | 2.1±0.12^c^ | 14 |
| ±1.41^d^10.2 | ±2.42^d^17.2 | ±2.41^d^27.4 | ±1.11^d^12.2 | ±1.14^d^15.6 | ±2.33^d^27.8 | ±0.14^d^0.8 | ±0.13^c^1.4 | ±0.35^c^2.2 | ±0.05^b^0.7 | ±0.09^c^1.2 | ±0.11^c^2.0 | 21 |
| 17.9±1.06^e^ | 29.9±3.03^e^ | 47.8±3.09^e^ | 17.8±1.22^e^ | 25.3±0.91^e^ | 43.1±2.12^e^ | 0.8±0.23^e^ | 1.9±0.63^d^ | 2.6±0.85^c^ | 0.8±0.01b^b^ | 1.3±0.15^c^ | 2.1±0.15^c^ | 28 |
| ±1.88^f^24.2 | .0±2.84^f^43 | ±3.76^f^67.2 | ±1.02^f^24.6 | ±2.32^f^36.8 | ±3.40^f^61.4 | ±0.16^c^0.9 | ±0.20^c^1.5 | ±0.22^c^2.4 | ±0.04^c^0.9 | ±0.10^c^1.1 | ±0.13^c^2.0 | 35 |
| 29.4±1.95^g^ | 54.4±5.24^g^ | 83.7±7.07^g^ | 28.8±1.46^g^ | 45.1±1.17^g^ | 73.9±2.55^g^ | 0.7±0.27^e^ | 1.5±0.41^c^ | 2.2±0.65^c^ | 0.6±0.13^d^ | 1.3±0.07^c^ | 1.9±0.18^c^ | 42 |
| ±2.41^h^37.9 | ±4.91^h^66.2 | ±7.52^h^104.1 | ±1.23^h^32.6 | ±2.05^h^53.0 | ±2.25^h^85.6 | ±0.10^c^0.9 | ±0.15^c^1.4 | ±0.15^c^2.3 | ±0.07^c^0.9 | ±0.05^bd^0.7 | ±0.11^bc^1.6 | 49 |
| 41.2±2.02^hi^ | 75.8±8.32^i^ | 117±10.35^i^ | 36.1±1.19^i^ | 58.4±1.35^hi^ | 94.6±2.2^hi^ | 0.9±0.32^c^ | 1.5±0.42^c^ | 2.4±0.71^c^ | 0.5±0.18^e^ | 0.8±0.13^d^ | 1.3±0.30^d^ | 56 |
| ±2.27^i^44.9 | ±8.16^i^77.3 | ±9.44^i^122.2 | ±1.44^ij^37.4 | ±1.19^i^59.4 | ±3.57^i^96.8 | ±0.12^e^0.7 | ±0.10^b^0.7 | ±0.12^d^1.4 | ±0.11^f^0.4 | ±0.14^e^0.3 | ±0.09^e^0.7 | 63 |
| 46.1±2.98^i^ | 79.8±9.1^i^ | 125.9±12.02^i^ | 41.2±1.04^j^ | 61.4±0.93^i^ | 102.6±1.77^i^ | 0.0±0.00^a^ | 0.0±0.00^a^ | 0.00±0.00^a^ | 0.2±0.06^g^ | 0.0±0.00^a^ | 0.2±0.06^f^ | 70 |
| 340.34 | 111.61 | 162.21 | 235.41 | 2531.30 | 1716.37 | 14.78 | 15.99 | 16.43 | 30.31 | 150.63 | 98.01 | F value |
| 0.0001 | 0.0001 | 0.0001 | 0.0001 | 0.0001 | 0.0001 | 0.0001 | 0.0001 | 0.0001 | 0.0001 | 0.0001 | 0.0001 | P value |

Values are the mean of three replicates ± SD.

Values with the same letter in the same column are insignificant (p≤0.05)

**Table S3.** Physico-chemical properties of untreated and pretreated substrate

| Pretreated | | | Untreated | | | RT (day) |
| --- | --- | --- | --- | --- | --- | --- |
| C/N | EC (dSm^-1^) | pH | C/N | EC (dSm^-1^)* | pH |  |
| 26.1±0.05^a^ | 3.2±0.03^a^ | 7.5±0.22^a^ | 28.8±0.03^a^ | 2.6±0.02^a^ | 7.1±0.33^a^ | 0 |
| ±0.07^ab^27.9 | ±0.05^ab^2.8 | ±0.12^ab^7.4 | ±0.04^a^25.1 | ±0.05^ab^3.4 | ±0.15^a^6.9 | 7 |
| 23.7±0.03^b^ | 3.6±0.30^b^ | 7.3±0.35^ab^ | 27.1±0.06^ab^ | 2.9±0.01^b^ | 7.0±0.21^a^ | 14 |
| ±0.06^c^25.0 | ±0.09^bc^3.1 | ±0.15^ab^7.0 | ±0.01^b^21.2 | ±0.02^b^3.8 | ±0.20^ab^6.7 | 21 |
| 19.4±0.06^c^ | 3.9±0.01^bc^ | 6.7±0.15^b^ | 24.1±0.04^b^ | 3.2±0.06^bc^ | 6.5±0.15^b^ | 28 |
| ±0.09^d^23.0 | ±0.06^c^3.3 | ±0.11^b^6.8 | ±0.05^bc^16.2 | ±0.05^bc^4.1 | ±0.11^b^6.5 | 35 |
| 14.8±0.01^de^ | 4.2±0.05^cd^ | 6.9±0.24^ab^ | 21.2±0.04^c^ | 3.4±0.03^c^ | 6.6±0.03^b^ | 42 |
| ±0.05^e^20.0 | ±0.11^cd^3.6 | ±0.25^ab^7.1 | ±0.02^cd^14.1 | ±0.02^cd^4.4 | ±0.10^ab^6.7 | 49 |
| 13.6±0.01^e^ | 4.5±0.01^cd^ | 7.0±0.30^ab^ | 19.2±0.07^cd^ | 3.6±0.05^cd^ | 6.9±0.23^ab^ | 56 |
| ±0.05^ef^18.4 | ±0.10^cd^3.6 | ±0.25^a^7.5 | ±0.05^d^13.0 | ±0.01^d^4.7 | ±0.17^c^7.3 | 63 |
| 12.3±0.08^f^ | 4.6±0.00^d^ | 7.8±0.15^a^ | 18.0±0.04^d^ | 3.7±0.03^d^ | 7.5±0.10^c^ | 70 |
| 41654.9 | 67.78 | 11.94 | 22737.32 | 359.43 | 9.78 | F value |
| 0.0001 | 0.0001 | 0.0003 | 0.0001 | 0.0001 | 0.0006 | P value |

Values are the mean of three replicates ± SD.

Values with the same letter in the same column are insignificant (p≤0.05)

*dSm^-1^ (decisiemens per metre) and l/KgVS (liter per kilogram volatile solids)
